# Myocardial fibrosis in rheumatic heart disease: emerging concepts and clinical implications

**DOI:** 10.3389/fcvm.2023.1230894

**Published:** 2023-07-19

**Authors:** Teuku Muhammad Haykal Putra, Rodrigo Rodriguez-Fernandez, Wishnu Aditya Widodo, Maria Elfiana, Sidhi Laksono, Quang Ngoc Nguyen, Jack Wei Chieh Tan, Jagat Narula

**Affiliations:** ^1^ Department of Cardiology and Vascular Medicine, Jakarta Heart Center, Jakarta, Indonesia; ^2^ International SOS, London, United Kingdom; ^3^ Research Unit, Jakarta Heart Center, Jakarta, Indonesia; ^4^ Faculty of Medicine, Universitas Muhammadiyah Prof. DR. Hamka, Tangerang, Indonesia; ^5^ Department of Cardiology, Hanoi Medical University, Hanoi, Vietnam; ^6^ Department of Cardiology, National Heart Centre Singapore, Singapore, Singapore; ^7^ Icahn School of Medicine at Mount Sinai, New York, NY, United States

**Keywords:** myocardial fibrosis, rheumatic heart disease, Group A Streptoccocus, cross reactivity, LV dysfunction

## Abstract

Rheumatic heart disease (RHD) remains a significant cardiovascular burden in the world even though it is no longer common in affluent countries. Centuries of history surrounding this disease provide us with a thorough understanding of its pathophysiology. Infections in the throat, skin, or mucosa are the gateway for Group A Streptococcus (GAS) to penetrate our immune system. A significant inflammatory response to the heart is caused by an immunologic cascade triggered by GAS antigen cross-reactivity. This exaggerated immune response is primarily responsible for cardiac dysfunction. Recurrent inflammatory processes damage all layers of the heart, including the endocardium, myocardium, and pericardium. A vicious immunological cycle involving inflammatory mediators, angiotensin II, and TGF-β promotes extracellular matrix remodeling, resulting in myocardial fibrosis. Myocardial fibrosis appears to be a prevalent occurrence in patients with RHD. The presence of myocardial fibrosis, which causes left ventricular dysfunction in RHD, might be utilized to determine options for treatment and might also be used to predict the outcome of interventions in patients with RHD. This emerging concept of myocardial fibrosis needs to be explored comprehensively in order to be optimally utilized in the treatment of RHD.

## Introduction

Myocardial fibrosis in rheumatic heart disease (RHD) has been well described in previous studies (1–3). We previously relied solely on myocardial biopsies to diagnose myocardial fibrosis. However, it has a significant rate of sampling error and render the prevalence of myocardial fibrosis difficult to evaluate. With the advance of cutting-edge technology, myocardial fibrosis may now be easily detected with high accuracy and precision (4). The existence of myocardial fibrosis is essential in understanding the development of RHD (3). Myocardial fibrosis in RHD is noteworthy because its presence poses more risk to the patients. Myocardial fibrosis is mainly responsible for left ventricular (LV) dysfunction in RHD (5). Myocardial fibrosis is also associated with poor outcomes after mitral valve surgery in RHD (6). On the other hand, it might play an important role in several therapeutic aspects. The presence of myocardial fibrosis, which is associated with LV dysfunction, might require adjustments to patients' medication (5, 7). Myocardial fibrosis also has a significant role in predicting the outcome of interventions. This modifies our understanding of selecting the best course of treatment for the patients (6). It is also predicted to be an important indicator to evaluate medication therapy (8).

Myocardial fibrosis in RHD was noticed decades ago (1, 2). However, its role in clinical settings has not been explored sufficiently (8). Research progress surrounding this area of expertise is relatively slow compared to other cardiovascular problems such as coronary artery disease or heart failure. This deduction is inferred from the significantly decreased number of published materials regarding RHD over the years (9). In addition, the known clinical significance of myocardial fibrosis in RHD is currently based on observational studies (5–7). Accordingly, examinations for myocardial fibrosis in RHD have not yet been recommended by any guidelines or expert opinions (10). This review aims to compile what is known about myocardial fibrosis in RHD and its future perspective in order to ignite more progress in this field.

## Rheumatic heart disease

Although it is no longer prevalent in developed countries, RHD is nevertheless recognized as a global significant cardiovascular burden (11–13). The prevalence of RHD varies across the world. It is commonly known that RHD can be found prevalently in developing Asian and African countries (13–17). RHD is found at a rate of 5.7 per 1,000 individuals in Sub-Saharan African countries and 1.8 per 1,000 individuals in Northern Africa (18). The prevalence of RHD in Indonesia is 5.3 per 1,000 individuals (19). It is crucial to acknowledge that these prevalence figures are derived from clinical screening in the population (15, 18, 19). When systematic echocardiography is used as a screening tool, the prevalence rates increase to 21.5–30.4 per 1,000 individuals as reported in Cambodia and Mozambique (15, 16). On the other hand, the prevalence of RHD in high-income countries (HIC) is rapidly decreasing and has been recorded at as low as 0.5 per 1,000 individuals (11, 15). Moreover, RHD cases in developing countries have been associated with severe valve disease at a much younger age (16, 20). Discrepancy in epidemiological data between developing and developed countries are reportedly due to better quality of life and access to healthcare resulting in a lower transmission rate of Group A Streptococcus (GAS) bacterial infection, the causative agent of RHD. This epidemiologic discrepancy is the reason why most publications and studies regarding this topic are relatively old and originated primarily from Asia and Africa (9, 12).

Rheumatic heart disease has been recognized for more than two centuries. Scientists had previously attempted to establish a link between manifestations of rheumatic fever (RF) and the presence of RHD. Unfortunately, the technology was not enough to properly investigate the pathophysiology concept of the disease. In 1904, Aschoff presented the first description of a distinct RHD lesion (21, 22). He was the first to report the pathological aspects of RHD by describing a nodule identified in the heart of patients with RHD, which would be later known as the Aschoff body. Given that the Aschoff body is derived from lymphatic vessels of the heart, he stated that the damage to the myocardium was secondary to the specific lesion in the connective tissue rather than due to destruction by what was previously described as rheumatic poison (21). This updated understanding confirmed the pathophysiology theory that RHD is the result of an exaggerated immune response to specific bacterial epitopes (21, 22).

**Figure 1:**
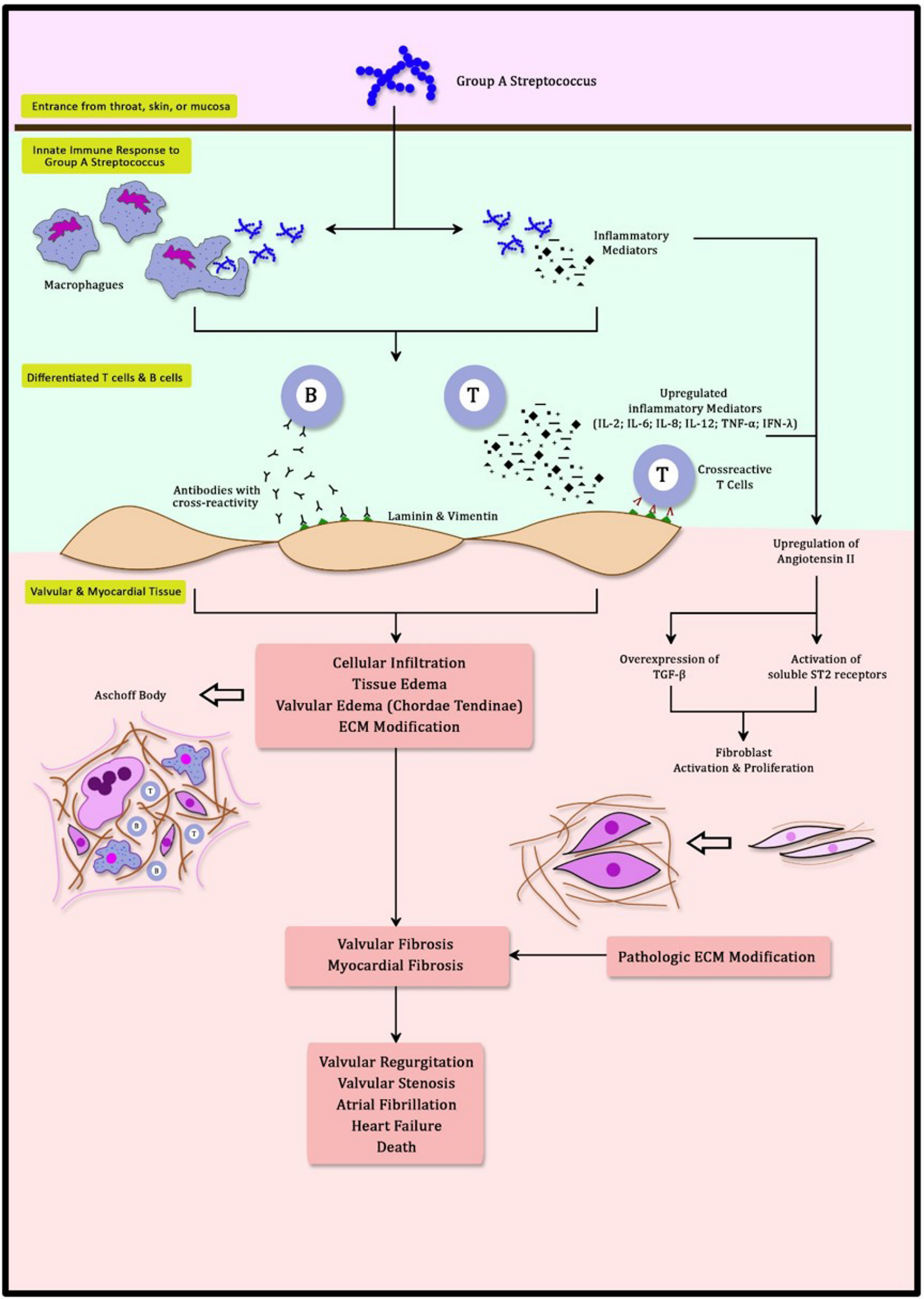
Schematic mechanism of molecular mimicry and myocardial fibrosis in rheumatic heart disease.

An exaggerated immune response is responsible for the development of RHD. Streptococcal infection in the throat, skin, or mucosa is the gateway for GAS to the body (23, 24). These first contacts of GAS activate innate immune responses involving neutrophils, dendritic cells, and macrophages (24, 25). Multiple inflammatory mediators excreted during this process, including cytokines and interleukins (IL), facilitate phagocytosis in order to eliminate the invading organisms (25). Furthermore, these inflammatory mediators (IL-2, IL-6, IL-8, IL-12, TNF-α, and IFN-γ) promote the differentiation of T cells and B cells (25, 26). B cells and T cells develop the ability to recognize GAS antigens through the amino acid sequence and its structural conformations (27, 28). This process triggers a mechanism known as molecular mimicry (12, 23, 24). M protein in GAS antigen shares a close resemblance to γ-helical coils structure found in both cardiac valvular and myocardial structures. N-Acetyl-β-D-glucosamine (GlcNAc) and myosin in the myocardium are the main targets of this cross-reactivity (23, 26, 29, 30). The Anti-GlcNAc and Anti-myosin complex is cytotoxic in nature and enhances both inflammation and fibrosis (3, 23, 26, 29). This causes T cells to induce cross-reactivity to both cardiac valvular and myocardial structures and generates the formation of autoantibodies (23). In healthy cardiac tissue, both GlcNAc and myosin are difficult to be accessed by the immune system (31). Anti-myosin leads to inflammation in the valve through its cross-reactivity with valve proteins laminin and vimentin (23, 26, 28, 30, 31). Cross-reactivity to these cell surface targets and extracellular matrix proteins (Collagens IV) is responsible for the infiltration of inflammatory mediators and antibodies to cardiac tissue (31). Furthermore, infiltration of these autoantibodies is enhanced with the activation of Vascular Cell Adhesion Molecule-1 (VCAM-1) (23, 26). These infiltrates can be detected further into the papillary muscle that contains myosin within its cardiomyocytes (26). This mechanism reinforced the long-held belief that the chordae tendineae are the most vulnerable cardiac structures to be affected by cross-reactivity antibodies (23). Excessively produced autoantibodies also upregulate inflammatory mediators, worsening inflammation specifically at the heart. This inflammation is granulomatous in nature and can be detected as Aschoff Body (26). Aschoff bodies are collections of interstitial inflammatory substances including lymphocytes, macrophages, B cells, Giant cells, and collagen necrosis (30). Recurrent GAS infection will recycle this process with a more pronounced inflammatory response, resulting in both valvular and myocardial dysfunction (Figure 1).

**Figure 2:**
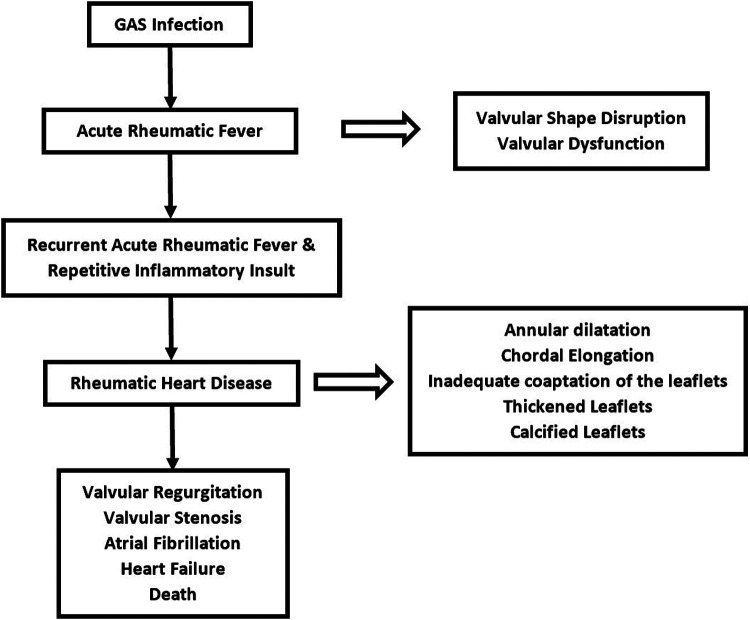
Natural history and progression of rheumatic heart disease.

The repeated inflammatory process of cross-reactivity between streptococcal antigen and tissue of the heart eventually leads to pathological dysfunction. This involves valve tissue, chordae tendineae, and myocardium. Repeated inflammation that occurred during multiple exposures to RF disrupts the shape and function of the valves (24). Cross-reactivity of antibodies generates inflammation at valve tissue incurring edema, cellular infiltration, and fibrinous vegetations (23). The initial lesion of the valve is typically characterized by annular dilatation and chordal elongation, leading to inadequate coaptation of the leaflets (23, 24, 26). Subsequently, It may be possible to identify tiny nodules at the coaptation sections of the valve leaflets (30, 32). Over time, the leaflets thicken with eventual deposition of fibrin on the cusps until the valves cannot maintain their physiological function (23). Parts of valves that are pathologically altered are leaflet commissures, leaflet cusps, and chordae tendineae (32). Commissural fusion and chordal shortening occur as a result of recurrent RF with repetitive valve scarring (23). Leaflet thickening and calcification are primarily due to the stress of chronic turbulence through a deformed valve (32). Mitral regurgitation (MR) is the first valve abnormality that appears shortly after RF presentation. Repeated RF incidents will accelerate the RHD progression over time. Mitral valve abnormalities will gradually develop into mitral stenosis (MS) (Figure 2) (23, 32). It is commonly accepted that it takes several years after the initial attack of RF for MS to manifest and it can take decades for MS to become visible in certain circumstances (32). This occurrence corresponds to epidemiological studies that revealed that rheumatic MR is dominant in younger patients and rheumatic MS becomes more prevalent with increasing age (12). The mitral valves are the most commonly afflicted valves in RHD, followed by the aortic valve (30). Valves with more hemodynamic stress, represented by their transvalvular pressure gradient, are more prone to acute valvulitis during RF presentation and may progress to RHD. This is mediated by tumor growth factor β-1 (TGF β-1), which epigenetically alters cells to promote more fibrosis and accelerate the advancement of RHD lesions (33, 34). Chronic overexpression of TGF β-1 in RHD cases stimulates fibroblasts to proliferate and creates pathological extracellular matrix components generating fibrosis in both valves and myocardium (34). Despite its predominance in valvular tissue, RHD is characterized pathologically by the involvement of all layers of the heart, including the pericardium, myocardium, and endocardium (23). The terms used to describe how RHD affects the heart are pancarditis, involving Aschoff bodies in the myocardium, fibrinous pericarditis, and valvulopathy (30). This pathological change is primarily responsible for fatal morbidities associated with RHD, such as heart failure, atrial fibrillation, stroke, and death (3).

The aforementioned autoimmunity cascade affected by GAS raises critical questions about the prevalence of RHD. If molecular mimicry is sufficient to initiate an autoimmune cascade, RHD prevalence should be significantly greater than the present estimate. Yet, only 1 individual out of 5,000 with GAS infections develops RF (31). This prevalence is actually an underestimated number because GAS infection is not easily detected even by various methods of examination. Children with reported detectable GAS infections do not account for all those with the disease. Nevertheless, this phenomenon denotes unique individual susceptibility to develop RHD when exposed to GAS infections. Individual genetic susceptibility and familial tendency to RHD have been widely studied (35). Approximately 14% of the population possess genes associated with an increased risk of RHD (31). Human Leukocyte-associated Antigen (HLA) class II genes located at chromosome 6 are believed to play a role in the development of both RF and RHD. This finding does not contradict the current understanding that these genes are responsible for regulating the immune response. Among these genes, HLA-DR7, DR4, and DR9 are the alleles that have consistently been found in the development of RHD (35).

## Myocardial fibrosis in RHD

Myocardial fibrosis is currently acknowledged to be a common finding in RHD. Multiple studies have identified the presence of myocardial fibrosis in patients with RHD. The first was published in 1968 by Shaper AG et al. This necropsy study found that 26 cases from 213 RHD patients were complicated by myocardial fibrosis (1). Another study was performed by Perennec et al. in 1980 in a different subset of population. Eleven samples of LV myocardial biopsies were studied from patients with isolated rheumatic MS who had corrective surgery. Four patients were identified with a moderate degree of myocardial fibrosis (36). Various degrees of myocardial fibrosis may develop in rheumatic MS. In 1999, Saraiva et al. observed extensive myocardial fibrosis in rheumatic MS with the fibrosis penetrating far into the myocardium (2).

The prevalence of myocardial fibrosis in RHD has not been established consistently, likely due to varying methods utilized to identify myocardial fibrosis. Older publications were using necropsy or myocardial biopsy samples, which are susceptible to sampling errors (1, 36). Recent use of cardiac MRI to identify myocardial fibrosis generates a relatively higher prevalence of myocardial fibrosis in RHD cases (5, 6). Nonetheless, the quality of the MRI image and the experience of the center were valid concerns for the possibility of bias. In Table 1, we present the prevalence of myocardial fibrosis in patients with RHD obtained from previous studies.

**Table 1:** Prevalence of myocardial fibrosis in rheumatic heart disease.

Author	Year	Location	Method of detection and sample characteristics	Sample size of RHD case	Prevalence
Shaper et al. (1)	1968	Uganda	Necropsy study.	213	12.2%
Perennec et al. (32)	1980	France	Myocardial biopsy in patients undergoing mitral valve surgery.	11	36.4%
Putra et al. (7)	2019	Indonesia	LGE protocol from cardiac MRI in patients undergoing mitral surgery.	47	91.5%

LGE, late gadolinium enhancement; MRI, magnetic resonance imaging.

Myocardial fibrosis is characterized by pathological manifestations of extracellular matrix (ECM) remodeling (37). It is marked by an increased collagen type I deposition and cardiac fibroblast activation (38). Regardless of the etiology, myocardial fibrosis leads to myocardial stiffness thereby causing cardiac dysfunction (38, 39). In RHD, myocardial fibrosis develops primarily due to an immunologic cascade triggered by cross-reactivity. Immunologic cascade, described as molecular mimicry, causes the upregulation of autoantibodies that exacerbate inflammation (24). This cascade involves autoantibodies and complement activation. Autoantibodies developed by cross-reactivity are the cornerstone of pathways leading to myocardial fibrosis. These autoantibodies develop a mechanism by overexpressing transforming growth factor β (TGF-β). Autoantibodies can directly cause injury at the endothelium and in the heart cell environment underneath (29). Consequently, the autoreactive innate immune system exacerbates tissue degradation, inflammation, neovascularization, and fibrosis in a negative cycle (3, 40). Angiotensin II has long been recognized as the primary stimulator of cardiac fibrosis (Figure 1) (41, 42). Angiotensin II generates fibrosis by stimulating TGF-β (41–44). This process is also observed in RHD (3). Angiotensin II also stimulates the soluble ST2 (sST2) decoy receptor, causing increased phosphorylation in the mitogen-activated protein kinase (MAPK) pathway. MAPK is a protein kinase that functions as a second messenger in response to extracellular stimuli. The MAPK pathway regulates gene expression, metabolism, cell proliferation, growth, differentiation, and survival (3, 45, 46). Activation of the MAPK pathway by TGF-β enhances ECM remodeling (37, 44, 46). Uncontrolled activity of TGF-β and the MAPK pathway will result in pathogenic fibrosis (43, 46).

There are several modalities we can use to detect myocardial fibrosis. Endomyocardial biopsy was the only available method to assess myocardial fibrosis prior to the development of more advanced techniques. Despite the advent of numerous novel diagnostic techniques, myocardial biopsies continue to be the gold standard for diagnosing myocardial fibrosis (4, 38). Its disadvantages include its invasive nature and a significant likelihood of sampling error in detecting localized fibrosis (4, 47). Cardiac MRI is a relatively recent modality used to detect fibrosis using the late gadolinium enhancement (LGE) protocol (4, 47, 48). LGE can easily detect and accurately analyze the extension of myocardial fibrosis in various diseases including RHD (4, 47, 49). The physiological basis of the LGE for identifying myocardial fibrosis is the combination of an increased volume of distribution for the contrast agent and a prolonged washout due to the decreased capillary density within the cardiac fibrotic tissue (4, 47). Description of myocardial fibrosis by LGE in patients with RHD is unique by its nature. It is frequently described as a patchwork pattern of myocardial fibrosis in the mid-myocardial part across every segment of the left ventricle (6, 50). Its appearance reflects the occurrence of myocardial fibrosis, which resulted from a non-ischemic condition (4, 50). T1 mapping is another novel protocol of cardiac MRI. This protocol is currently being developed and calibrated in recent studies (47, 48). Publications regarding the use of T1 mapping protocol in patients with RHD are still scarce (47, 48, 51). Another approach for detecting myocardial fibrosis is the ST2 biomarker. Soluble ST2 is a member of the IL 1 receptor family and it plays an important role in both the inflammatory response and the fibroproliferative mechanism. Its role as a decoy receptor for IL-33 attenuates its counterpart's (ST2 ligand) beneficial effect of reducing fibrosis. A higher level of soluble ST2 serum level is associated with increased myocardial fibrosis (3, 52). Other potential laboratory biomarkers for myocardial fibrosis are galectin-3 and procollagen (52).

## Clinical importance of myocardial fibrosis in RHD

### Myocardial fibrosis and LV dysfunction

Left ventricular dysfunction is common in RHD (53, 54) and manifests itself abruptly as a reduced left ventricular ejection fraction (LVEF). The prevalence of reduced LVEF in RHD varies greatly. Much of the research studying this area of interest is allegedly obsolete because most of the studies were several decades old due to the declining prevalence of RHD. In addition, most of the studies were also performed on a small number of subjects (54). Table 2 summarizes the various reported prevalence of reduced LVEF in RHD (5, 6, 20, 49, 55–58). Furthermore, LV dysfunction can be detected by speckle tracking echocardiography at an earlier stage of the disease before LVEF is compromised (59).

**Table 2:** Prevalence of reduced left ventricular ejection fraction in rheumatic heart disease.

Author	Year	Location	Sample characteristics	Sample size of RHD cases	Prevalence of reduced LVEF
Gash et al. (52)	1983	USA	Isolated rheumatic mitral stenosis patients.	16	31.3%
Lee et al. (53)	1990	Taiwan	Isolated rheumatic mitral stenosis patients.	15	40%
Choi et al. (45)	1995	USA	Isolated rheumatic mitral stenosis patients.	36	50%
Surdacki et al. (54)	1996	Poland	Isolated rheumatic mitral stenosis patients in sinus rhythm.	39	30.8%
Shikano et al. (55)	2003	Japan	Isolated rheumatic mitral stenosis patients.	33	21%
Elen et al. (6)	2017	Indonesia	Severe rheumatic mitral stenosis patients.	18	44.4%
Putra et al. (7)	2019	Indonesia	RHD patients undergoing mitral valve surgery.	47	21.3%
Rudiktyo et al. (18)	2022	Indonesia	All RHD patients were assessed by echocardiography in a tertiary national hospital.	2,333	15.1%

RHD, rheumatic heart disease; LVEF, left ventricular ejection fraction.

Reduced LVEF is known to occur in rheumatic MR due to the process of LV remodeling produced by myocardial stretch and increased filling pressure. Nevertheless, reduced LVEF was once a puzzling aspect of rheumatic MS because it was believed that pathological conditions only affected the valve, and the left ventricle was spared with no consequences (54, 60). The new concept of myocardial fibrosis in RHD explained why reduced LVEF can still be found in isolated rheumatic MS (7). Prior to the widespread recognition of myocardial fibrosis in RHD, this condition was referred to as “myocardial factor” (54, 60). A large number of studies explored the cause of LV dysfunction in rheumatic MS. Klein et al. highlighted all possible factors for RHD to develop LV dysfunction (54). These included reduced filling of LV, myocardial fibrosis, wall motion abnormalities, reduced LV compliance due to chronic decrease of preload, increased afterload, altered interaction between LV and RV, atrial fibrillation, and concomitant diseases (53, 54).

The first study explaining the association of LV dysfunction with myocardial fibrosis was published in 1973. Horwitz et al. identified kinetic abnormalities specifically in the anterior and posterior portions of the left ventricle in patients with RHD. This aberrant LV movement might have resulted from myocardial fibrosis formation at the papillary muscles of the mitral valve (61). Furthermore, Lee YS et al. conducted a microscopic pathology study on patients with RHD in 1990 and observed that RHD complicated with LV dysfunction exhibited more extensive loss of myofibrils due to disproportion or myofibril degeneration (56). A recent study exploring this issue was written by Elen et al. in 2017. Among 18 patients with severe rheumatic MS, it was concluded that those with a greater amount of myocardial fibrosis had a significantly decreased LVEF (5). Among multiple contributing factors to develop LV dysfunction in rheumatic MS, myocardial fibrosis is considered to be an important element (53, 54).

**Figure 3:**
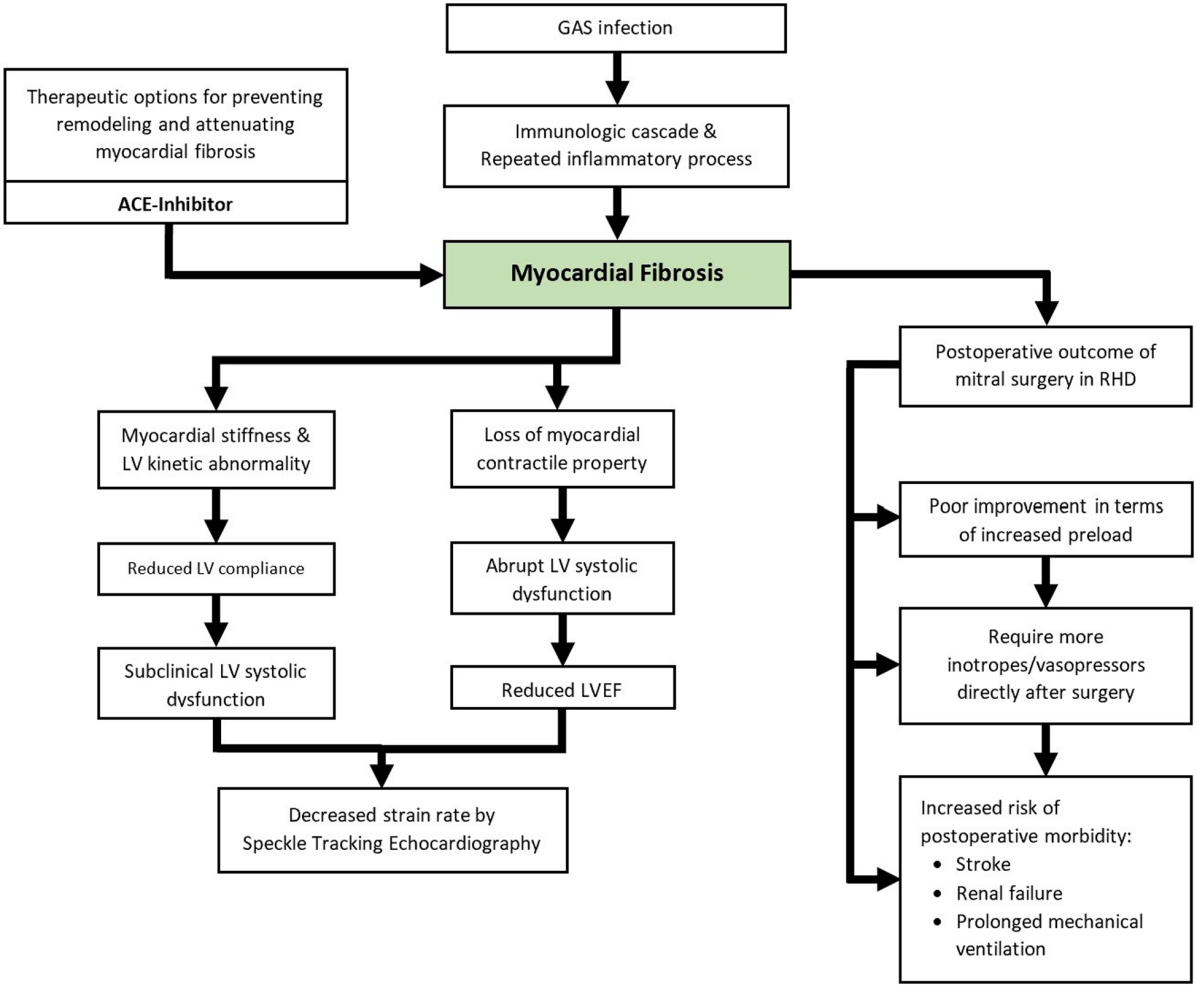
Schematic illustration of the currently known clinical impacts of myocardial fibrosis in RHD.

Deteriorated LV performance may be detected before LVEF is compromised by strain rate measurement from speckle tracking echocardiography (Figure 3). A small amount of myocardial fibrosis may not modify LVEF, but it will impair LV performance modestly and manifest as subclinical LV systolic dysfunction (7, 59). This slight change in LV function can be detected by using strain rate measurement from speckle tracking echocardiography with good reproducibility (59). This applies to multiple causes of myocardial fibrosis, including ischemic heart disease, aortic stenosis, aortic regurgitation, and hypertrophic cardiomyopathy. Numerous studies have investigated the use of speckle tracking echocardiography in detecting disturbances in LV performance among patients with RHD (7, 59, 62). In 2011, Bilen et al. described impaired LV function among patients with rheumatic MS measured by global longitudinal strain rate irrespective of the severity of the MS (62). Another speckle tracking study by Younan et al., conducted in 2015, showed significantly lower longitudinal strain and strain rate in moderate and severe rheumatic MS compared to its control healthy group (59). In 2019, Soesanto et al. investigated the association between myocardial fibrosis quantified by LGE protocol in cardiac MRI and global longitudinal strain rate by speckle tracking echocardiography in 36 patients with rheumatic MS who were scheduled for mitral surgery. It showed a moderate correlation between both variables. It concluded that a higher volume of myocardial fibrosis was associated with a more diminished LV performance (7).

### Myocardial fibrosis and the outcome of cardiac surgery

Numerous earlier studies have investigated the factors of poor surgical prognosis in RHD. Factors that appeared to be significant were RV dilatation, poor RV function, LA dilatation, and pulmonary hypertension (63–66). However, subsequent research has shown that these parameters are not associated with postoperative morbidity (6). This improvement may be the result of improved surgical technique and better ICU care (67).

Myocardial fibrosis is an important predictor of clinical outcomes after cardiac surgery. It is observed in patients with coronary artery disease (CAD), as reported by Kancharla et al. in 2016. Patients with a greater scar burden, as detected by LGE protocol, had worse long-term survival after coronary artery bypass graft (CABG) surgery (68). Similar results were reported by Chaikriangkrai et al. in a different subset of the population. A higher incidence of adverse clinical outcomes was found in patients with myocardial fibrosis following mitral valve repair in chronic MR of ischemic origin after approximately 1-year follow-up (69). This has also been seen in non-ischemic myocardial fibrosis. Barone-Rochette et al. established that the presence of myocardial fibrosis was an independent predictor of survival in patients with aortic stenosis who had aortic valve replacement surgery (70).

The impact of myocardial fibrosis in RHD on postoperative outcomes has been explored previously by several studies (Figure 3) (2, 6, 49, 50). Substantial findings were described by Putra et al. in 2019. It was observed that patients with postoperative morbidity presented with a significantly larger volume of myocardial fibrosis measured by LGE protocol prior to surgery. More extensive myocardial fibrosis was associated with an increased risk of postoperative morbidity after mitral valve surgery (6). The influence of myocardial fibrosis on the dimensions and geometrical changes of the left ventricle after mitral valve surgery in patients with RHD was examined in a 2020 study. Increased LV preload was observed exclusively among patients with less than 5% myocardial fibrosis, as indicated by increased postoperative LV End-Diastolic Diameter (EDD) (50). Increased LV EDD is in line with increased stroke volume and cardiac output, especially after the removal of a restrictive flow in the mitral valve (71). A smaller amount of myocardial fibrosis was associated with favorable improvements in LV geometry (50). It should be noted that these studies were describing the immediate outcomes of the surgery and long-term prognosis has not been explored.

### Myocardial fibrosis and medication therapy

Cardiac remodeling characterized by myocardial fibrosis is a part of the cardiovascular continuum. This chain of events happens to various causes of cardiovascular disease such as ischemic heart disease, hypertension, valvular heart disease, and inflammatory conditions of the heart. Interventions at any point within the continuum may modify disease progression and prevent further remodeling and fibrosis (72). Medical management of myocardial fibrosis has been focusing on preventing the progression of the disease. However, there are indications that myocardial fibrosis reversal may occur with specific medications (42, 73). The Renin Angiotensin Aldosterone (RAA) system played a significant role in advancing any cardiovascular disease into cardiac remodeling or myocardial fibrosis (42, 72). It resolves around the actions attributed to angiotensin II, which is a potent activator of cardiac fibroblasts (42). Drugs affecting the RAA system have been known to have significant effects in modifying cardiac remodeling and treating myocardial fibrosis. Angiotensin-converting enzyme (ACE) inhibitors and angiotensin II receptor blockers (ARB) have demonstrated such capacity, as observed in several clinical studies (42, 72–74). ACE-inhibitors are considered to be potentially beneficial, especially for RHD, not only in interfering with the RAA system but also for attenuating myocardial fibrosis by inhibiting TGF-β signaling (42, 75).

Myocardial fibrosis in RHD is greatly influenced by angiotensin II, TGF-β, and the MAPK signaling pathway, as discussed previously (3, 34). By inhibiting angiotensin II, ACE inhibitors are thought to be effective at targeting this pathway (3, 8, 75). ACE inhibitors reduce inflammation and fibrosis by lowering IL-6 and TNF-α (76), and also trigger the apoptosis of cardiac fibroblasts, which generate pathological ECM components for myocardial fibrosis (3, 34). These effects of ACE inhibitors are the highlight of their role as anti-remodeling and antifibrosis treatment (3). Even though this mechanism has been seen in other causes of myocardial fibrosis, the role of ACE inhibitors in RHD cases has not yet been confirmed.

ACE inhibitors are the drug of choice to treat heart failure in valvular regurgitation (10, 77). However, its effectiveness in mitral stenosis is unclear (8, 78). ACE inhibitors were thought to induce hypotension due to their effect of afterload reduction in a mechanically obstructive flow of the mitral valve, thus increasing the transvalvular gradient (8, 50, 54, 77). This hypothesis is apparently not true, according to a study conducted in 2005 by Chockalingam. The study was a randomized controlled trial with 109 patients with RHD that analyzed the safety of enalapril in rheumatic MS. It was concluded that enalapril was well-tolerated in patients with rheumatic MS even at higher doses. In addition to its safety profile, enalapril also improved functional status and exercise capacity at 30 days follow-up (77). Current guidelines for valvular heart disease do not mention ACE-Inhibitors as a useful medication therapy for rheumatic MS even though its safety has been established (10, 77). Even though rheumatic MS manifests clinically as heart failure, there are limited data exploring its medical treatments. This is because most heart failure trials exclude valvular heart disease in their population (54). An ongoing randomized controlled trial by Ambari et al. is currently underway to determine the efficacy of ACE inhibitors in treating rheumatic MS (8, 13).

There are other drugs known for their effectiveness as medications for myocardial fibrosis that exhibit significant clinical benefits. These drugs are β-blockers, mineralocorticoid receptor antagonists, statins, TGF-β inhibitors, and colchicine (38, 73, 74). Some of these drugs are widely being used in heart failure treatment while showing antifibrosis properties. In addition, β-blockers and mineralocorticoid receptor antagonists are commonly prescribed for rheumatic MS patients presenting with heart failure signs and symptoms (10). Other approaches currently being investigated include stem-cell therapy and recombinant growth factors treatment (38). Despite its various mechanisms in treating myocardial fibrosis, none of these therapeutic modalities has been developed to be utilized in RHD.

## Future perspective of myocardial fibrosis in RHD

**Figure 4:**
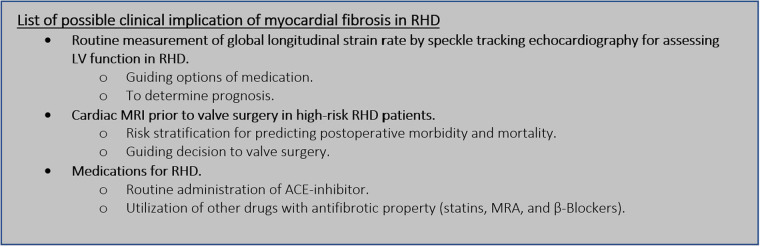
Future perspective of myocardial fibrosis in RHD. LV, left ventricle; RHD, rheumatic heart disease; MRI, magnetic resonance imaging; ACE, angiotensin converting enzyme; MRA, mineralocorticoid receptor antagonist.

Evolving research regarding myocardial fibrosis in the field of RHD encourages clinicians to adopt a more comprehensive approach to treating RHD. Utilizing recent discoveries with significant clinical impact in our daily practice will benefit patients with RHD. Further advantages can be gained when more research for clinical benefit is conducted in this area of expertise. Future research can guide both medical and surgical programs for the patients (Figure 4).

Speckle tracking echocardiography is used to analyze subtle changes in LV performance before LVEF is compromised (7, 59). It was commonly thought that inflammatory insult had little effect on the left ventricle in RHD (54, 60). As a result, there was little interest in assessing LV function thoroughly beyond the LVEF measurement. Recent studies on myocardial fibrosis in patients with RHD have provided clinicians with additional evidence of the limitations of LVEF measurement in reflecting LV function. It has been established that global longitudinal strain rates from speckle tracking echocardiography are worse in patients with RHD than in a normal population (7, 59, 62). A larger area of myocardial fibrosis is likewise associated with a worse global longitudinal strain rate (7). The clinical significance of a worse global longitudinal strain rate in RHD patients has not yet been determined. Hence, there is currently no recommendation to routinely perform speckle tracking echocardiography in RHD other than to confirm subtle changes in LV function. Such a recommendation might be proposed when its clinical impact is discovered. It is to be expected that future investigations will be focusing on the clinical value of a poor strain rate from speckle tracking echocardiography. Information regarding the long-term progression of the disease based on strain rate findings would give a crucial perspective on the timing of valve surgery.

The prognostic implications of myocardial fibrosis in RHD had been established in patients planned for surgery. More extensive myocardial fibrosis, as measured by LGE from cardiac MRI, significantly leads to worse postoperative morbidity (6). This insight might be used to guide the decision for mitral valve surgery for certain patients with RHD. In patients with ischemic heart disease, LGE findings from cardiac MRI were utilized to determine the risk and benefits of surgical treatment (68, 78). The degree of transmurality determined by the LGE protocol can be used to establish the viability of specific regions of the left ventricle and whether or not the patient requires revascularization procedures (78–80). Transmurality quantification is not appropriate for quantifying myocardial fibrosis in RHD due to the fact that it is not subendocardial in nature (6, 50). The degree of myocardial fibrosis in RHD is based on the extension of its volume and it can be classified as less than 5% and ≥5%. Less than 5% myocardial fibrosis implies a more favorable improvement of LV geometry after mitral surgery (50). However, its association with long-term prognosis has not been explored yet. Information about long-term morbidity and mortality after valve surgery in RHD based on myocardial fibrosis findings is necessary to firmly determine recommendations regarding decisions to perform surgery. This issue needs more validation and confirmation before being implemented in daily clinical practice.

Medications for RHD recommended by current guidelines reside in treating the congestion nature of the disease (10). The presence of myocardial fibrosis in RHD uncovers a new perspective on drug therapy. Unfortunately, medications specifically targeted to treat myocardial fibrosis in RHD have not been explored. This is due to the fact that myocardial fibrosis in RHD is a relatively new concept. The antifibrosis property of some medications may be advantageous in the treatment of RHD (3, 8, 75). ACE inhibitors are debatable medications for treating myocardial fibrosis in RHD due to concerns about their safety (75, 77). Ongoing randomized controlled trials are being conducted to assess the efficacy of ACE inhibitors in reducing myocardial fibrosis (8). The impact of treatment on myocardial fibrosis in RHD is unclear without the result of such research. The results may significantly alter the fundamental treatment for RHD.

## Conclusions

Myocardial fibrosis is a common finding in patients with RHD. It is fundamentally generated by an exaggerated immune response to the GAS antigen because of its cross-reactivity with cardiac valvular and myocardial structures. The presence of myocardial fibrosis and its impact on patients with RHD have been confirmed by multiple studies. Myocardial fibrosis appears to be important in various clinical aspects. Myocardial fibrosis is responsible for LV dysfunction in RHD cases. It also plays a significant role in predicting clinical outcomes of interventions in patients with RHD. The presence of myocardial fibrosis uncovers different approaches to medication therapy for RHD. Medications with antifibrotic properties such as ACE inhibitors might hold a potential role in the treatment of RHD. Future perspective regarding this issue requires additional research to determine its clinical utility in daily practice.

## References

[1] Shaper A Hutt M Coles R. Necropsy study of endomyocardial fibrosis and rheumatic heart disease in Uganda 1950-1965. Brit Heart J. (1968) 30:391–401. 10.1136/hrt.30.3.391 5651254 PMC487634

[2] Saraiva L Carneiro R Arruda M Brindeiro D Lira V. Mitral valve disease with rheumatic appearance in the presence of left ventricular endomyocardial fibrosis. Arq Bras Cardiol. (1999) 72:330–2. 10.1590/s0066-782/1999000300006 10513044

[3] Ambari AM Setianto B Santoso A Radi B Dwiputra B Susilowati E Angiotensin converting enzyme inhibitors (ACEIs) decrease the progression of cardiac fibrosis in rheumatic heart disease through the inhibition of IL-33/sSTS2. Front Cardiovasc Med. (2020) 7:1–9. 10.3389/fcvm.2020.00115 32850979 PMC7399157

[4] Mewton N Liu C Croisille P Bluemke D Lima J. Assessment of myocardial fibrosis with cardiovascular magnetic resonance. J Am Coll Cardiol. (2011) 57(8):891–903. 10.1016/j.jacc.2010.11.013 21329834 PMC3081658

[5] Elen E Atmadikoesoemah CA Kasim M. Effect of myocardial fibrosis on left ventricular function in rheumatic mitral stenosis: a preliminary study with cardiac magnetic resonance. Indonesian J Cardiol. (2017) 38:202–6. 10.30701/ijc.v38i4.785

[6] Putra TMH Sukmawan R Elen E Atmadikoesoemah CA Desandri DR Kasim M. Prognostic value of late gadolinium enhancement in postoperative morbidity following mitral valves surgery in rheumatic mitral stenosis. Int J Angiol. (2019) 28(4):237–44. 10.1055/s-0039-1693457 31787822 PMC6882674

[7] Soesanto M Desandri DR Haykal TM Kasim M. Association between late gadolinium enhancement and global longitudinal strain in patients with rheumatic heart disease. Int J Cardiovasc Imaging. (2019) 35(5):781–9. 10.1007/s10554-018-1511-1 30556113

[8] Ambari AM Setianto B Santoso A Radi B Dwiputra B Susilowati E Randomised controlled trial into the role of ramipril in fibrosis reduction in rheumatic heart disease: the RamiRHeD trial protocol. BMJ Open. (2021) 11:e048016. 10.1136/bmjopen-2020-048016 34518254 PMC8438922

[9] Carapetis JR. Rheumatic heart disease in developing countries. N Engl J Med. (2007) 357(5):439–41. 10.1056/NEJMp078039 17671252

[10] Vahanian A Beyersdorf F Praz F Milojevic M Baldus S Bauersachs J 2021 ESC/EACTS guidelines for the management of valvular heart disease. Eur Heart J. (2022) 43:561–632. 10.1093/eurheartj/ehab395 34453165

[11] Watkins DA Johnson CO Colquhoun SM Karthikeyan G Beaton A Bukhman G Global, regional and national burden of rheumatic heart disease, 1990-2015. N Eng J Med. (2017) 377:713–22. 10.1056/NEJMoa1603693 28834488

[12] Carapetis JR McDonald M Wilson NJ. Acute rheumatic fever. Lancet. (2005) 366:155–68. 10.1016/S0140-6736(05)66874-2 16005340

[13] Soesanto AM. Editorial: new challenges with the management of rheumatic heart disease. Front Surg. (2022) 9:1030172. 10.3389/fsurg.2022.1030172 36303846 PMC9592842

[14] Bhaya M Panwar S Beniwal R Panwar RB. High prevalence of rheumatic heart disease detected by echocardiography in school children. Echocardiography. (2010) 27(4):448–53. 10.1111/j.1540-8175.2009.01055.x 20345448

[15] Lung B. Mitral stenosis still a concern in heart valve disease. Arch Cardiovasc Dis. (2008) 101(10):597–9. 10.1016/j.acvd.2008.09.003 19056064

[16] Marijon E Ou P Celermajer DS Ferreira B Mocumbi AO Jani D Prevalence of rheumatic heart disease detected by echocardiographic screening. N Engl J Med. (2007) 357(5):470–6. 10.1056/NEJMoa065085 17671255

[17] Damasceno A Mayosi BM Sani M Ogah OS Mondo C Ojji D The causes, treatment, and outcome of acute heart failure in 1006 Africans from 9 countries. Arch Intern Med. (2012) 172(18):1386–94. 10.1001/archinternmed.2012.3310 22945249

[18] Nkomo VT. Epidemiology and prevention of valvular heart diseases and infective endocarditis in Africa. Heart. (2007) 93(12):1510–9. 10.1136/hrt.2007.118810 18003682 PMC2095773

[19] Rodriguez-Fernandez R Amiya R Wyber R Widdodo W Carapetis J. Rheumatic heart disease among adults in a mining community of Papua, Indonesia: findings from an occupational cohort. Heart Asia. (2015) 7:1–5. 10.1136/heartasia-2015-010641 26294934 PMC4537650

[20] Rudiktyo E Wind A Doevendans P Siswanto BB Cramer MJ Soesanto AM. Characteristic of patients with rheumatic heart disease in a national referral hospital in Indonesia. Med J Indones. (2022) 31:178–85. 10.13181/mji.oa.226150

[21] Wedum BG McGuire JW. Origin of the aschoff body. Ann Rheum Dis. (1963) 22:127–41. 10.1136/ard.22.3.127 13999453 PMC1007339

[22] Roberts WC Virmani R. Aschoff bodies at necropsy in valvular heart disease: evidence from an analysis of 543 patients over 14 years of age that rheumatic heart disease, at least anatomically, is a disease of the mitral valve. Circulation. (1978) 57:803–7. 10.1161/01.cir.57.4.803 630691

[23] Cunningham MW. Rheumatic fever, autoimmunity and molecular mimicry: the streptococcal connection. Int Rev Immunol. (2014) 33(4):314–29. 10.3109/08830185.2014.917411 24892819 PMC4669348

[24] Carapetis JR Beaton A Cunningham MW Guilherme L Karthikeyan G Mayosi BM Acute rheumatic fever and rheumatic heart disease. Nat Rev Dis Primers. (2016) 2:15084. 10.1038/nrdp.2015.84 27188830 PMC5810582

[25] Fieber C Kovarik P. Responses of innate immune cells to group A Streptococcus. Front Cell Infect Microbiol. (2014) 4(140):1–7. 10.3389/fcimb.2014.00140 25325020 PMC4183118

[26] Cunningham MW. Streptococcus and rheumatic fever. Curr Opin Rheumatol. (2012) 24(4):408–16. 10.1097/BOR.0b013e32835461d3 22617826 PMC3645882

[27] McNamara C Zinkernagel AS Macheboeuf P Cunningham MW Nizet V Ghosh P. Coiled-coil irregularities and instabilities in group A streptococcus M1 are required for virulence. Science. (2008) 319(5868):1405–8. 10.1126/science.1154470 18323455 PMC2288698

[28] Fae KC da Silva DD Oshiro SE Tanaka AC Pomerantzeff PMA Douay C Mimicry in recognition of cardiac myosin peptides by heart-intralesional T cell clones from rheumatic heart disease. J Immunol. (2006) 176(9):5662–70. 10.4049/jimmunol.176.9.5662 16622036

[29] Galvin JE Hemric ME Ward K Cunningham MW. Cytotoxic mAb from rheumatic carditis recognizes heart valves and laminin. J Clin Invest. (2000) 106(2):217–24. 10.1172/JCI7132 10903337 PMC314302

[30] Yanagawa B Butany J Verma S. Update on rheumatic heart disease. Curr Opin Cardiol. (2016) 31:162–8. 10.1097/HCO.0000000000000269 26731292

[31] Root-Bernstein R. Rethinking molecular mimicry in rheumatic heart disease and autoimmune myocarditis: laminin, collagen IV, CAR, and B1AR as initial targets of disease. Front Pediatr. (2014) 2:85. 10.3389/fped.2014.00085 25191648 PMC4137453

[32] Selzer A Cohn KE. Natural history of mitral stenosis: a review. Circulation. (1972) 45(4):878–90. 10.1161/01.cir.45.4.878 4552598

[33] Karthikeyan G Fung E Foo RS. Alternative hypothesis to explain disease progression in rheumatic heart disease. Circulation. (2020) 142:2091–4. 10.1161/CIRCULATIONAHA.120.050955 33253001

[34] Kim L Kim DK Yang WI Shin DH Jung IM Park HK Overexpression of transforming growth factor-β1 in the valvular fibrosis of chronic rheumatic heart disease. J Korean Med Sci. (2008) 23:41–8. 10.3346/jkms.2008.23.1.41 18303197 PMC2526480

[35] Guilherme L Kalil J Cunningham M. Molecular mimicry in the autoimmune pathogenesis of rheumatic heart disease. Autoimmunity. (2006) 39(1):31–9. 10.1080/08916930500484674 16455580

[36] Perennec J Herreman F Ameur A Degeorges M Hatt PY. Ultrastructural and histological study of left ventricular myocardium in mitral stenosis. Basic Res Cardiol. (1980) 75(2):353–64. 10.1007/BF01907583 7396813

[37] Fan D Takawale A Lee J Kassiri Z. Cardiac fibroblasts, fibrosis and extracellular matrix remodeling in heart disease. Fibrogenesis Tissue Repair. (2012) 5(15):1–13. 10.1186/1755-1536-5-15 22943504 PMC3464725

[38] Morfino P Aimo A Castiglione V Galvez-Monton C Emdin M Bayes-Genis A. Treatment of cardiac fibrosis: from neuro-hormonal inhibitors to CAR-T cell therapy. Heart Failure Rev. (2023) 28(2):555–69. 10.1007/s10741-022-10279-x PMC9553301 36221014

[39] Jellis C Martin J Narula J Marwick TH. Assessment of nonischemic myocardial fibrosis. J Am Coll Cardiol. (2010) 56(2):89–97. 10.1016/j.jacc.2010.02.047 20620723

[40] Watkins DA Beaton AZ Carapetis JR Karthikeyan G Mayosi BM Wyber R. Rheumatic heart disease worldwide: JACC scientific expert panel. J Am Coll Cardiol. (2018) 72:1397–416. 10.1016/j.jacc.2018.06.063 30213333

[41] Travers JG Kamal FA Robbins J Yutzey KE Blaxall BC. Cardiac fibrosis: the fibroblast awakens. Circ Res. (2016) 118:1021–40. 10.1161/CIRCRESAHA.115.306565 26987915 PMC4800485

[42] Hinderer S Schenke-Layland K. Cardiac fibrosis—a short review of causes and therapeutic strategies. Adv Drug Deliv Rev. (2019) 146:77–82. 10.1016/j.addr.2019.05.011 31158407

[43] Meng XM Nikolic-Paterson DJ Lan HY. TGF-β: the master regulator of fibrosis. Nat Rev Nephrol. (2016) 12(6):325–38. 10.1038/nrneph.2016.48 27108839

[44] Gui T Sun Y Shimokado A Muragaki Y. The roles of mitogen-activated protein kinase pathways in TGF-β-induced epithelial-mesenchymal transition. J Signal Transduct. (2012) 2012:289243. 10.1155/2012/289243 22363839 PMC3272823

[45] Cargnello M Roux PP. Activation and function of the MAPKs and their substrates, the MAPK-activated protein kinases. Microbiol Mol Biol Rev. (2011) 75:50–83. 10.1128/MMBR.00031-10 21372320 PMC3063353

[46] Rose BA Force T Wang Y. Mitogen-activated protein kinase signaling in the heart: angels versus demons in a heart-breaking tale. Physiol Rev. (2010) 90(4):1–63. 10.1152/physrev.00054.2009 20959622 PMC3808831

[47] Ambale-Venkatesh B Lima JAC. Cardiac MRI: a central prognostic tool in myocardial fibrosis. Nat Rev Cardiol. (2015) 12(1):18–29. 10.1038/nrcardio.2014.159 25348690

[48] Podleniskar T Delgado V Bax JJ. Cardiovascular magnetic resonance imaging to assess myocardial fibrosis in valvular heart disease. Int J Cardiovasc Imaging. (2018) 34(1):97–112. 10.1007/s10554-017-1195-y 28642994 PMC5797565

[49] Choi EY Yoon SJ Lim SH Choi BW Ha JW Shin DH Detection of myocardial involvement of rheumatic heart disease with contrast-enhanced magnetic resonance imaging. Int J Cardiol. (2006) 113(2):36–8. 10.1016/j.ijcard.2006.04.013 16759716

[50] Putra TMH Sukmawan R Desandri DR Atmadikoesoemah CA Elen E Kasim M. Left ventricular dimension after mitral valve surgery in rheumatic mitral stenosis: the impact of myocardial fibrosis. J the Univ Heart Ctr. (2020) 15(3):119–27. 10.18502/jthc.v15i3.4222 PMC7827121 33552207

[51] Li S Wang S Yu J Sun J Cheng W Liu J Myocardial extracellular volume assessed by cardiovascular magnetic resonance may predict adverse left ventricular remodeling in rheumatic heart disease after valvular surgery. Quant Imaging Med Surg. (2022) 12(4):2487–97. 10.21037/qims-21-678 35371927 PMC8923869

[52] Sharim J Daniels LB. Soluble ST2 and soluble markers of fibrosis: emerging roles for prognosis and guiding therapy. Curr Cardiol Rep. (2020) 22(41):1–8. 10.1007/s11886-020-01288-z 32430626

[53] Buyukkaya S Buyukkaya E Arslan S Aksakal E Sevimli S Gundogdu F Evaluation of left ventricular long-axis function in cases of rheumatic pure mitral stenosis with atrial fibrillation. Tex Heart Inst J. (2008) 35(1):22–7. 18427646 PMC2322880

[54] Klein AJP Carroll JD. Left ventricular dysfunction and mitral stenosis. Heart Fail Clin. (2006) 2(4):443–52. 10.1016/j.hfc.2006.09.006 17448431

[55] Gash AK Carabello BA Cepin D Spann JF. Left ventricular ejection performance and systolic muscle function in patients with mitral stenosis. Circulation. (1983) 67(1):148–54. 10.1161/01.cir.67.1.148 6847794

[56] Lee YS Lee CP. Ultrastructural pathological study of left ventricular myocardium in patients with isolated rheumatic mitral stenosis with normal or abnormal left ventricular function. Jpn Heart J. (1990) 31(4):435–48. 10.1536/ihj.31.435 2232163

[57] Surdacki A Legutko J Turek P Dudek D Zmudka K Dubiel JS. Determinants of depressed left ventricular ejection fraction in pure mitral stenosis with preserved sinus rhythm. J Heart Valve Dis. (1996) 51(1):1–9. 8834717

[58] Shikano M Nakatani S Kim J Hanatani A Hashimura K Yasumura Y Impaired left ventricular systolic function in mitral stenosis. J Cardiol. (2003) 42(2):75–9. 12964517

[59] Younan H. Role of two-dimensional strain and strain rate imaging in assessment of left ventricular systolic function in patients with rheumatic mitral stenosis and normal ejection fraction. Egyptian Heart J. (2015) 67(3):193–8. 10.1093/ehjci/jeu253

[60] Carabello BA. Modern management of mitral stenosis. Circulation. (2005) 112(3):432–7. 10.1161/CIRCULATIONAHA.104.532498 16027271

[61] Horwitz LD Mullins CB Payne RM Curry GC. Left ventricular function in mitral stenosis. Chest. (1973) 64(5):609–14. 10.1378/chest.64.5.609 4750333

[62] Bilen E Kurt M Tanboga IH Kaya A Isik T Ekinci M Severity of mitral stenosis and left ventricular mechanics: a speckle tracking study. Cardiology. (2011) 119(2):108–15. 10.1159/000330404 21912124

[63] Ye Y Desai R Vargas Abello LM Rajeswaran J Klein AL Blackstone EH Effects of right ventricular morphology and function on outcomes of patients with degenerative mitral valve disease. J Thorac Cardiovasc Surg. (2014) 148(5):2012–20. 10.1016/j.jtcvs.2014.02.082 24698557

[64] Reed D Abbott RD Smucker ML Kaul S. Prediction of outcome after mitral valve replacement in patients with symptomatic chronic mitral regurgitation. The importance of left atrial size. Circulation. (1991) 84(1):23–34. 10.1161/01.cir.84.1.23 2060099

[65] Dokhan A El-Raouf M Ibrahim I Abdellatif M. Evaluation of early outcomes after mitral replacement in rheumatic heart patients with pulmonary hypertension. Menoufia Med J. (2016) 29:674–9. 10.4103/1110-2098.198753

[66] Severino ES Petrucci O Vilarinho KA Lavagnoli CFR Filho LMS Oliveira PPM Late outcomes of mitral repair in rheumatic patients. Rev Bras Cir Cardiovasc. (2011) 26(4):559–64. 10.5935/1678-9741.20110045 22358270

[67] Cesnjevar RA Feyrer R Walther F Mahmoud FO Lindemann Y von der Emde J. High-risk mitral valve replacement in severe pulmonary hypertension–30 years experience. Eur J Cardiothorac Surg. (1998) 13(4):344–51. 10.1016/s1010-7940(98)00042-6 9641330

[68] Kancharla K Weissman G Elagha AA Kancherla K Samineni S Hill PC Scar quantification by cardiovascular magnetic resonance as an independent predictor of long-term survival in patients with ischemic heart failure treated by coronary artery bypass graft surgery. J Cardiovasc Magn Reson. (2016) 18(45):1–9. 10.1186/s12968-016-0265-y 27430331 PMC4950709

[69] Chaikriangkrai K Lopez-Mattei JC Lawrie G Ibrahim H Quinones MA Zoghbi W Prognostic value of delayed enhancement cardiac magnetic resonance imaging in mitral valve repair. Ann Thorac Surg. (2014) 98(5):1557–63. 10.1016/j.athoracsur.2014.06.049 25240782

[70] Barone-Rochette G Piérard S De Meester de Ravenstein C Seldrum S Melchior J Maes F Prognostic significance of LGE by CMR in aortic stenosis patients undergoing valve replacement. J Am Coll Cardiol. (2014) 64(2):144–54. 10.1016/j.jacc.2014.02.612 25011718

[71] Sengupta SP Amaki M Bansal M Fulwani M Washimkar S Hofstra L Effects of percutaneous balloon mitral valvuloplasty on left ventricular deformation in patients with isolated severe mitral stenosis: a speckle-tracking strain echocardiographic study. J Am Soc Echocardiogr. (2014) 27(6):639–47. 10.1016/j.echo.2014.01.024 24637055

[72] Frangogiannis NG. Cardiac fibrosis. Cardiovasc Res. (2021) 117(6):1450–88. 10.1093/cvr/cvaa324 33135058 PMC8152700

[73] Spoladore R Falasconi G Fiore G Di Maio S Preda A Slavich M Cardiac fibrosis: emerging agents in preclinical and clinical development. Expert Opin Investig Drugs. (2021) 30(2):153–66. 10.1080/13543784.2021.1868432 33356660

[74] Webber M Jackson SP Moon JC Captur G. Myocardial fibrosis in heart failure: antifibrotic therapies and the role of cardiovascular magnetic resonance in drug trials. Cardiol Ther. (2020) 9:363–76. 10.1007/s40119-020-00199-y 32862327 PMC7584719

[75] Chockalingam A Venkatesan S Dorairajan S Chockalingam V Subramaniam T Jaganathan V Safety and efficacy of enalapril in multivalvular heart disease with significant mitral stenosis—SCOPE-MS. Angiology. (2005) 56(2):151–8. 10.1177/000331970505600205 15793604

[76] Ferrario CM. Cardiac remodelling and RAS inhibition. Ther Adv Cardiovasc Dis. (2016) 10(3):162–71. 10.1177/1753944716642677 27105891 PMC5192558

[77] Abareshi A Norouzi F Asgharzadeh F Beheshti F Hosseini M Farzadnia M Effect of angiotensin-converting enzyme inhibitor on cardiac fibrosis and oxidative stress status in lipopolysaccharide-induced inflammation model in rats. Int J Prev Med. (2017) 8(69):1–7. 10.4103/ijpvm.IJPVM_322_16 28966758 PMC5609356

[78] Carpenter JP Pennell DJ Prasad SK. Chapter 28: Heart failure—CMR to assess viability. In: Zamorano JL Bax J Knuuti J Sechtem U Lancellotti P Badano L, editors. The ESC textbook of cardiovascular imaging. UK: Oxford University press (2015). p. 380–95.

[79] Loffler AI Kramer CM. Myocardial viability testing to guide coronary revascularization. Interv Cardiol Clin. (2018) 7(3):355–65. 10.1016/j.iccl.2018.03.005 29983147 PMC6044453

[80] Chan J Khafagi F Young AA Cowan BR Thompson C Marwick TH. Impact of coronary revascularization and transmural extent of scar on regional left ventricular remodelling. Eur Heart J. (2008) 29:1608–17. 10.1093/eurheartj/ehn247 18556718

